# Humeral Head‐Split Fracture in Two Dogs

**DOI:** 10.1111/vru.70001

**Published:** 2025-01-06

**Authors:** Ingrid Isaac, Ian Faux, Dylan Neil Clements, Wilfried Mai, Amy Kapatkin, Tobias Schwarz

**Affiliations:** ^1^ Royal (Dick) School of Veterinary Studies and Roslin Institute The University of Edinburgh Roslin UK; ^2^ Department of Clinical Sciences and Advanced Medicine University of Pennsylvania, School of Veterinary Medicine Section of Radiology Philadelphia Pennsylvania USA; ^3^ Department of Surgical & Radiological Sciences School of Veterinary Medicine, University of California‐Davis Davis California USA

**Keywords:** canine, CT, head‐split fracture, glenohumeral joint, proximal humerus fracture, radiograph, Salter‐Harris, shoulder

## Abstract

Two skeletally immature female dogs were each investigated for chronic weight‐bearing thoracic limb lameness. The first patient was lame for 2 months following a tumble whilst playing, and the second patient had been intermittently lame since 3 weeks of age. In both cases, radiographic examination of the shoulder revealed fissuring of the caudal humeral head consistent with an incomplete proximal humeral Salter‐Harris type IV fracture with an Enoki‐mushroom‐like appearance of the caudal fragment, where two heads rise from a common stem. There was secondary neoarthrosis of the caudal humeral head fragment with the glenoid rim of the scapula. Humeral head‐split fracture is an unusual fracture pattern that rarely occurs in skeletally immature patients, and conservative management appears to result in reasonable short‐term outcomes. The role of early detection and surgical intervention remains unknown.

## 1 I Signalment, History, and Clinical Findings

### 1.1 Case 1

**FIGURE 1:**
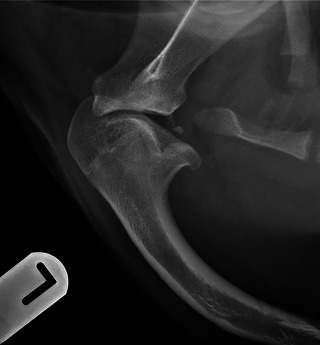
Mediolateral radiograph of the left shoulder of a 9‐month‐old Labrador retriever (case 1). Note the caudal elongation of the humeral head extending beyond the glenoid and its flattened appearance. The humeral head split appearance resembles two Enoki mushrooms growing out of one stem. A large osseous body and other small fragments are present within the caudal articular space, detached from the flattened caudal glenoid rim of the scapula. There is additionally premature closure of the proximal humeral physis and cranial bowing of the proximal and mid‐humeral diaphysis.

A 9‐month‐old, 24 kg, female Labrador Retriever was presented for a referral orthopedic consultation following a 2‐month duration left thoracic limb lameness, which started after a tumble whilst playing. On orthopedic examination, the left glenohumeral joint was painful on manipulation with moderate crepitus and a reduced range of motion on flexion and extension. A radiographic examination of the shoulders and elbows was performed at the referring veterinarian's practice, which revealed an abnormally flattened caudal aspect of the left humeral head (Figure 1).

### 1.2 Case 2

A 5‐month‐old, 14.9 kg, female Australian Shepherd dog was presented for a referral orthopedic consultation following a long‐standing waxing, and waning history of weight‐bearing right thoracic limb lameness. The lameness had first been noticed by the breeder at 3 weeks of age, which improved following a 2‐week course of nonsteroidal anti‐inflammatory drugs and broad‐spectrum antibiotics. One month prior to presentation, the lameness became persistent. On orthopedic examination, the patient displayed a grade 1/10 lameness, a moderate degree of muscular atrophy, and a shortening of the right thoracic limb.

## 2 I Imaging, Diagnosis, and Ouctomes

**FIGURE 2:**
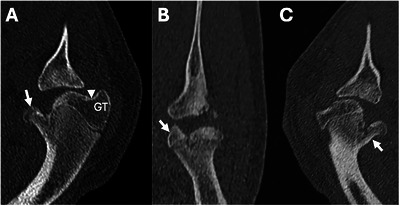
CT images of the left proximal humerus of case 1 with (A) an oblique‐plane reconstructed image depicting the greater tubercle (GT), the bicipital groove (arrowhead), and the caudomedial humeral head fragment (arrow). B, Dorsal‐plane reconstructed image showing the medially displaced fragment (arrow) and (C) near‐sagittal‐plane reconstructed image showing the caudomedial fragment attached to the metaphyseal bone (arrow).

Case 1 underwent a CT examination of the shoulders and elbows, performed under sedation, with the patient positioned in ventral recumbency and with the neck laterally flexed to avoid attenuation of the X‐ray beam, without the administration of intravenous iodinated contrast medium. The study was acquired with a 64‐row multidetector CT scanner (Somatom Definition AS Siemens, Erlangen, Germany). The technical settings were 120 kV, 0.4 s rotation time, 48 × 3 mm collimation configuration, and 512 × 512 matrix at a pitch of 0.85, with the mAs being adjusted by the automatic exposure control system (Care Dose 4D, Siemens Medical Solutions, International). Slice thickness was set at 0.6 mm, and images were obtained using a bone algorithm (Siemens proprietary iterative kernel U80u; window width 4000 HU, window level 700 HU). Extending from the proximal epiphysis to the proximal metaphysis of the left humerus, there was an incomplete fracture of the caudomedial aspect of the humeral head with an epiphyseal fragment still attached to the metaphyseal bone. This resulted in a split appearance of the humeral head, resembling an Enoki mushroom where two fruiting bodies rise from a common stem (Figure 2). The caudal humeral head was flattened and shallow, and there was concurrent flattening of the articular surface of the glenoid fossa. A separate osseous fragment was noted within the articular space, with a matching osseous defect in the caudal glenoid. The articular surface of the humeral head was irregularly marginated with multiple subchondral defects. Given the atypical appearance of the humeral fracture, the authors determined the osseous density of the greater tubercle of the humerus of the affected dog and a signalment‐matched control dog using the CT‐osteodensitometry software (Osteo, Siemens) with calibration phantom (model number 8783219, Siemens, Munich, Germany). A mean trabecular bone mineral density of 243.8 mg calcium hydroxyapatite/ml was calculated for case 1. In a 7‐month‐old female Labrador retriever, CT scanned for elbow lameness, and with no evidence of generalized bone disease, the mean trabecular bone mineral density was 249.5 mg calcium hydroxyapatite/mL. An incomplete proximal humeral Salter‐Harris type IV fracture and secondary glenoid neoarthrosis were diagnosed. There was a concurrent caudal glenoid Salter‐Harris type III fracture with an associated joint mouse and an ipsilateral left humeral condylar fissure, which was stabilized the following day with transcondylar screw fixation.

On the 9‐week recheck appointment, the patient exhibited a grade 2/10 lameness on the left thoracic limb, with moderate muscle atrophy and reduced extension of the shoulder, but not requiring ongoing analgesia. Radiographs of the elbows and shoulders were acquired, which did not reveal progressive remodeling of the left glenohumeral joint changes.

**FIGURE 3:**
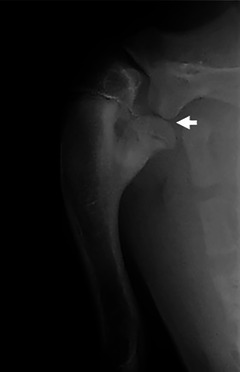
Mediolateral radiograph of the right shoulder of a 5‐month‐old Australian shepherd dog (case 2). Note the head‐split appearance of the proximal humerus and the neoarthrosis of the caudal humeral head fragment with the flattened glenoid rim of the scapula (arrow).

Case 2 underwent a radiographic examination of the right shoulder. On the mediolateral projection, there was a split appearance of the humeral head, with the caudal fragment still attached to the metaphyseal bone, resembling an Enoki mushroom (Figure 3). The cartilage‐covered caudal humeral head fragment was displaced beyond the caudal margin of the glenoid fossa of the scapula, resulting in neoarthrosis. Additionally, there was premature closure of the proximal humeral physis, resulting in shortening of the diaphysis. Mild lateral bowing of the proximal humeral diaphysis was noted, resulting in mild valgus deformity. An unwitnessed traumatic injury with a vertical shearing force impacting the humeral head was suspected, resulting in an incomplete Salter‐Harris type IV fracture. The incomplete fracture resulted in a neoarthrosis of the cartilage‐covered caudal humeral head fragment with the caudal glenoid rim of the scapula. The orthopedic surgeon discussed surgical intervention after skeletal maturity was reached to correct the length discrepancy of the radius and ulna, preventing the worsening of the degenerative joint disease affecting the shoulder. No further follow‐up information was available.

## 3 Discussion

This is the first report describing the radiographic and CT features of humeral head‐split fractures in dogs. The humerus is the least commonly fractured long bone in the dog, with an incidence between 5.4 and 7.7% [1, 2]. Proximal fractures are the least common humeral fractures in dogs [2]. This is most likely due to the large volume of the humeral head, its enclosed location within the glenoid fossa, and its close location to the trunk [2, 3].

The mechanism of injury behind proximal humeral fractures is likely related to the tension and compression forces exerted onto the proximal humerus: the supraspinatus muscle contraction creates a tensile force onto the greater tubercle during the swing phase of the gait, whilst the humeral head is compressed against the glenoid during the weight‐bearing phase [4]. If a lateral shearing force is inflicted against the proximal humerus in a skeletally immature dog, it may result in fissuring of the humeral head. Depending on the force of impact, it may result in an incomplete fracture, leading to the observed double humeral head appearance resembling a Japanese Enoki mushroom with two fruiting bodies growing out of the stem.

In humans, proximal humeral fractures are rare and typically associated with two different groups: young, skeletally healthy males involved in high‐energy trauma (e.g., high‐speed road traffic accidents, epileptic seizures, horse riding falls, motorcycle accidents) and, more commonly, elderly women, involved in low‐energy traumatic events (e.g., simple fall on to the shoulder when walking, hiking), due to impaired bone quality with limited regenerative potential such as in osteoporosis [5, 6]. Both patients in this report were skeletally immature, in agreement with the increased susceptibility of immature bones to this mechanism of injury. An important consideration is the time of occurrence. In dogs, reported proximal humeral physeal closure time varies between seven and a half and 18 months of age [7, 8]. In case 2, the traumatic insult took place in a very early phase of growth, leading to the premature closure of the proximal humeral physis and growth arrest of the affected limb. In patient one, the injury occurred at a later stage of skeletal growth, and no significant discontinuation of longitudinal growth was noted. Underlying development bone conditions, such as osteochondrosis and shoulder dysplasia, may act as predisposing factors.

Both the described cases resulted in incomplete fractures with only a partial disruption of the vascularization to the humeral head. The primary vascular supply to the humeral head in the dog is provided by the cranial and caudal circumflex arteries, which are branches of the axillary artery. Around the physis, these arteries develop numerous anastomoses supplying the epiphyseal, metaphyseal, and capsular structures. In fractures involving the complete displacement of the humeral head, there is a risk for humeral head ischemia and avascular necrosis, representing a therapeutical challenge [5]. Other risk factors for ischemia include the length of the fracture line involving the metaphysis, the integrity of the periosteal hinge, and fracture comminution [9, 10]. In both presented cases, a partial disruption of the vascular supply to the metaphysis was suspected, which, alongside the persistent weight‐bearing forces exerted on the glenohumeral joint, may have conditioned the healing process, resulting in a persistent fissure pattern.

Injury to the brachial plexus can be associated with such lesions, including axillary nerve neuropraxia, which may occur as a consequence of the displacement of a large osseous fragment and hematoma formation [6]. None of the presented patients exhibited neurological deficits. Surgery is the preferred treatment for most articular fractures. However, the present study documents two cases in which conservative management was pursued, with case 1 having short‐term follow‐up demonstrating a reasonable functional outcome. It is unknown if these partial injuries would have benefitted from surgical intervention.

The diagnosis was achieved with orthogonal radiographs in both cases, which suggests that this modality alone is sufficient to establish the existence of an articular fracture of the humeral head. Additional CT scanning is valuable when evaluating the fracture pattern and alignment of the fragments, the necessity for surgical reduction and fixation, and accurate surgical planning.

## 4 Conclusion

Humeral head‐split fractures can be recognized in the dog with characteristic radiographic and CT features of a partial split appearance of the humeral head with a distinct Enoki‐mushroom‐like appearance. Recognition of this condition is relevant in raising awareness of the radiographic appearance and a better understanding of the outcomes and treatment options available.

## List of Author Contributions

### Category 1

(a) Conception and design: Schwarz, Isaac
(b) Acquisition of data: Schwarz, Isaac, Faux, Mai, Kapatkin
(c) Analysis and Interpretation of data: Schwarz, Isaac, Faux, Clements

### Category 2

(a) Drafting the article: Isaac, Schwarz
(b) Revising the article for intellectual content: Schwarz, Isaac, Faux, Mai, Kapatkin, Clements

### Category 3

(a) Final approval of the completed article: Schwarz, Isaac, Faux, Mai, Kapatkin, Clements

### Category 4

(a) Agreement to be accountable for all aspects of the work in ensuring that questions related to the accuracy or integrity of any part of the work are appropriately investigated and resolved: Schwarz, Isaac, Faux, Mai, Kapatkin, Clements

## Ethics Statement

The University of Edinburgh's ethical guidelines for case reports were followed when undertaking this work. A high standard (best practice) of veterinary care was followed and informed client consent was given.

## Equator Network Disclosure

The authors followed the Strobe‐Vet network.

## Conflicts of Interest

The authors declared no conflicts of interest.

## Publication Disclosure

This work was presented as a poster at the ECVDI Conference, in Athens Greece, September 19–21, 2024.
